# Linking Life Aspirations to Functional Medical Conditions: A Goal Contents Theory Perspective

**DOI:** 10.3390/ijerph22101582

**Published:** 2025-10-17

**Authors:** Adam Neufeld, Emma L. Bradshaw

**Affiliations:** 1 Department of Family Medicine, University of Calgary, Calgary, AB T2N 4N1, Canada; 2 Institute for Positive Psychology and Education, Australian Catholic University, North Sydney, NSW 2060, Australia; emma.bradshaw@acu.edu.au

**Keywords:** extrinsic goals, functional somatic symptoms, intrinsic goals, motivational health links, self-determination theory

## Abstract

Psychological and motivational factors are implicated in various medical conditions, yet the link between physical health and life aspirations, as defined in Self-Determination Theory (SDT), remains underexplored. To address this gap and advance theory, we conducted a preliminary investigation of associations between aspirations and self-reported symptoms across five functional medical conditions—gastroesophageal reflux disease (GERD), irritable bowel syndrome (IBS), headaches, sleep disturbances, and sexual dysfunction. We surveyed 392 Canadian medical patients (Mage = 42.8 years, SD = 12.7, 50.5% women, 82.1% white, 75.3% with higher education) to assess whether the relative importance, likelihood, and attainment of intrinsic (e.g., personal growth, relationships, community, health) and extrinsic (e.g., wealth, fame, image) aspirations were associated with symptoms. Consistent with hypotheses, greater relative prioritization of intrinsic goals was linked to fewer symptoms—especially sleep disturbance—while extrinsic aspirations were associated with increased symptoms, particularly GERD. Sociodemographic factors, such as age, gender, education, religiosity, and subjective financial status, also showed associations with goal orientations and symptom burden, broadly aligning with SDT predictions. Findings highlight the potential relevance of people’s personal goals in patient-centered care for functional conditions and underscore the need for further research exploring mechanisms and moderators of these effects.

## 1. Introduction

Functional symptoms—persistent physical complaints without clear organic pathology [1]—are among the most common reasons for seeking primary care. These conditions, often termed functional somatic disorders, are heterogenous and involve complex interactions between biological, psychological, and social factors that contribute to distress and functional impairment [2]. Common examples include irritable bowel [3], chronic fatigue [4], and sexual dysfunction [5]. Despite their prevalence and burden on healthcare systems [6], they remain notoriously difficult to treat when biomedical explanations fall short. The extent to which psychological states, such as motivation and striving, relate to these conditions remains poorly understood.

A promising framework for addressing that gap is Self-Determination Theory (SDT) [7], a comprehensive theory of human motivation that emphasizes the conditions that support or thwart basic psychological needs for autonomy, competence, and relatedness. Within SDT, Goal Contents Theory [8] distinguishes between intrinsic aspirations or goals (e.g., personal growth, close relationships, community contribution, and physical health) and extrinsic aspirations (e.g., financial success, image, popularity). Extensive research shows that intrinsic goals are associated with greater well-being, while focusing on extrinsic goals predicts greater ill-being. These differential effects appear to arise because intrinsic goals directly satisfy basic psychological needs, whereas extrinsic goals only indirectly satisfy and often directly frustrate them [9,10]. The intrinsic–extrinsic pattern has been replicated across diverse populations, ages, and cultural contexts [11]. A recent meta-analysis also confirmed that these associations hold across different temporal dimensions of goal pursuit—how important goals are to people, how likely they are to be achieved, and whether they have already been attained [8].

Goal Contents Theory research has focused on the psychological outcomes of intrinsic and extrinsic goals, such as happiness or depression. However, there is growing recognition that goal orientation also relates to physical health outcomes. For example, intrinsic aspirations have been linked to higher rates of smoking cessation [12], sustained exercise [13,14,15,16] and other healthy lifestyle behaviors [17]. Conversely, extrinsic aspirations have been associated with increased physical symptoms such as headaches, stomach discomfort, and musculoskeletal pain [18,19,20]. Most of these studies were conducted among student samples; however, they relied on correlations that did not account for overall levels of goal striving. This analytic choice limits the conclusions because the theory holds that extrinsic goals are not ‘bad’ per se but rather become increasingly costly as they become more dominant within the broader pattern of aspirations. Accounting for relative prioritization when assessing intrinsic and extrinsic goals in relation to physical health is therefore crucial for interpretability and generalizability.

Equally important to interpreting the associations between aspirations and outcomes is recognizing that those links may not be uniform across demographic groups. Demographic factors shape people’s goal orientations and may therefore shine unique and novel light on their health implications. Although Goal Contents Theory posits that the pathways from intrinsic aspirations to well-being and from extrinsic aspirations to ill-being are universal, individual and cultural differences may influence how strongly certain goals are valued and feel attainable [11]. For example, age and education are positively associated with goal clarity and shifts towards life priorities that are more intrinsic, specific, and time-bound [21], meaning age and education could interact with aspirations in relation to physical health outcomes. Further, gender socialization has led men to view status- and income-related goals as both more valuable and more attainable, whereas women place greater value on relational and community goals and see high-status aspirations as less achievable. How those differences affect the links between aspirations and functional symptoms is still unknown [18,22,23]. Socioeconomic status, including both objective and subjective income, has also been linked to differences in goal content, with lower income levels often associated with more extrinsic striving [24,25]. Ethnic identity and religiosity may also moderate aspiration–outcome links, because some cultural and religious frameworks can lead people to value community and self-transcendence, which may buffer or amplify downstream effects on health [26,27]. Examining how these sociodemographic factors interact with goal orientations in relation to functional symptoms thus provides a more nuanced understanding of how aspiration orientations relate to physical health.

Taken together, the evidence suggests that, despite expected demographic variation in magnitude, focusing on extrinsic goals undermines well-being and fosters ongoing need frustration, which is reliably associated with increases in stress-related biomarkers [28,29]. In contrast, intrinsic aspirations build well-being and need satisfaction, which support better nervous system and immune function [30]. We extend this line of reasoning and evidence to examine whether the relative prioritization of intrinsic versus extrinsic goals is associated with functional symptoms in five domains: gastroesophageal reflux disease (GERD), irritable bowel syndrome (IBS), headaches, sleep disturbance, and sexual dysfunction, and how goal orientations interact with important demographic variables in relation to these outcomes. Although our dataset does not include biomarkers, the evidence outlined here provides a plausible pathway linking life aspirations to stress-sensitive functional symptoms. Assessing the basic association was our first aim, and the identification of mediators will be an important part of future research.

In the present research, we tested two primary hypotheses:

1. That greater relative prioritization of intrinsic goals would be negatively associated with symptoms across all five domains.
2. That greater relative prioritization of extrinsic goals would be positively associated with symptoms of these same conditions.

This study offers four key contributions: (1) the examination of functional symptoms not previously tested in relation to intrinsic and extrinsic aspirations, extending the literature on personal goals and physical health; (2) an assessment using relative centrality scores as opposed to raw correlational methods, (3) the inclusion of all three temporal domains of aspiring—importance, likelihood, and attainment, which permits a more fine-grained view of how aspirations relate to functional symptoms; and (4) the inclusion of important demographic moderators, which adds nuance and generalizability to the associations. Together, these contributions represent a meaningful step toward integrating motivation science into biopsychosocial models of care.

## 2. Materials and Methods

### 2.1. Participants and Procedures

A total of 645 patients from an urban medical clinic in Calgary, Alberta, were invited to complete an online survey, open for eight weeks from October to December 2023. Participants were 18–65 years old, cognitively fit, could read and understand basic English, and had an active email address. For recruitment, flyers were posted throughout the clinic, and patients were either verbally asked by their physician (author AN) if they were interested in participating, or they volunteered spontaneously. Those who showed interest were sent an email invitation from a third-party address containing information about the study, a consent form, and a link to the survey tool. The recruitment approach was approved by the University of Calgary’s Conjoint Health Research Ethics Board and aligned with national ethical guidelines [31], which permit physician-led recruitment when participation is voluntary, anonymous, and does not impact clinical care. The survey was not separately pilot-tested, as all instruments were previously validated. However, its format and flow were refined through prior patient surveys in the same clinic to optimize clarity, length, and completion rates.

The survey contained demographic questions and six previously validated scales (see Measures). We chose these self-report measures because they are widely used and accepted in the healthcare literature. The demographic items asked about age, gender (woman, man, non-binary), education (less than high school, high school, college/university or higher), income ($49,000 and under, $50,000–$99,000, $100,000 and over), subjective financial status (from 1 = much worse to 10 = much better, compared to other Canadians), ethnicity (White, Black, Latino/Hispanic, Asian, Indigenous, Native Hawaiian/Pacific Islander, Other), and religiosity (not at all important, somewhat important, very important). All responses were optional, and surveys were anonymous.

### 2.2. Ethics

This research received approval from the University of Calgary Conjoint Human Research Ethics Board (REB # 23-1282).

### 2.3. Measures

Life Aspirations. The Aspiration Index [18] assessed participants’ intrinsic and extrinsic life goals. The measure captures seven goal categories: four intrinsic (relationships, personal growth, community, and health), and three extrinsic (wealth, fame, and image/status). We used a shortened 14-item version [32] to minimize participant burden. In this version, each aspiration category was represented by two items and participants rated their importance, likelihood of future attainment, and current degree of attainment, using a scale from 1 (not at all) to 7 (very). Example items include, “to be a very wealthy person” (extrinsic), and “to have good friends that I can count on” (intrinsic). Higher scores indicated more of each aspect. The shortened scale has been shown to have strong psychometric properties, including among Canadian medical patients [33]. In the current study, Cronbach’s alphas for the overall intrinsic and extrinsic goal importance, likelihood, and attainment factors showed acceptable internal consistency (0.71 to 0.82).

Gastroesophageal Reflux. The Gastroesophageal Reflux Disease Impact Scale (GIS) has eight items and measures the frequency of reflux symptoms (acid-related, chest pain, and extra-esophageal) and their impact on sleep, work, meals, social occasions, and use of non-prescription drugs over the past six weeks. Studies have shown that patients find the GIS simple to complete and that it has strong psychometric properties [34]. Participants rated their degree of symptom frequency and severity—for example, “a burning sensation in your chest or behind your breastbone” or “regurgitation or acid taste in your mouth”—using a 4-point scale, ranging from 1 (none of the time) to 4 (all the time). Higher scores represented more frequent and bothersome GERD symptoms.

Irritable Bowel Syndrome. The Birmingham IBS Symptom Questionnaire has 11 items and measures the frequency and severity of IBS symptoms over the last four weeks. It has three subscales: constipation, diarrhea, and abdominal pain. An example item is, “During the last 4 weeks, how often have you been troubled by discomfort or pain in your abdomen?” with similar questions about constipation and diarrhea. Studies have shown that the scale is easy to interpret and that it is a reliable and valid measure of IBS [35]. Each question is answered using a 6-point Likert scale, ranging from 0 (none of the time) to 5 (all the time), where higher dimension or summary scores indicate more frequent and severe symptoms of IBS.

Headache. The Headache Impact Test (HIT-6) measures the frequency and impact of headaches on a person’s functioning. It is considered a valid tool to evaluate the impact of primary and secondary headaches, including acute and chronic migraine [36,37]. The questionnaire consists of six items that are rated on a scale from 1 (never) to 5 (always), where columns 1 (never) through 5 (always) are worth 6, 8, 10, 11, and 13 points each, respectively. A few example items are, “When you have headaches, how often is the pain severe?” and “In the past 4 weeks, how often have you felt too tired to do work or daily activities because of your headaches?” Scores can therefore range from 36 to 78. A total score is calculated by summing the column scores where higher scores indicate more frequent and bothersome headaches.

Sleep Disturbance. The Global Sleep Assessment Questionnaire (GSAQ) contains 11 items and is considered one of the best screening tools for sleep disorders for the general population [38]. It measures symptoms of insomnia (inability to fall and/or remain asleep), parasomnias (abnormal movements, talking, emotions and actions during sleep, such as sleep terrors, sleepwalking, and sleep paralysis), restless leg syndrome (uncontrollable urges to move the legs during sleep), and sleep apnea (periodic episodes where breathing transiently stops and sleep is disrupted), and how these symptoms impact mood and lifestyle over the last 4 weeks. An example item was, “During the past 4 weeks, how often did you have difficulty falling asleep, staying asleep, or feeling poorly rested in the morning?” Patients indicate how frequently and severely they experience these issues on a scale from 0 (never) to 3 (always). Higher scores thus indicate more frequent and bothersome sleep disturbance. The GSAQ scoring assesses each diagnosis separately and items are summed for an overall score.

Sexual Dysfunction. The Arizona Sexual Experiences Scale (ASEX) has five items and measures sexual functioning. It is considered a reliable instrument for identifying and quantifying sexual dysfunction across a range of populations [39]. The ASEX applies to both men and women and captures four main components based on their experiences over the last week: self-reported sex drive, vaginal lubrication or penile erection, ability to reach orgasm, and satisfaction from orgasm. A few example items are, “How strong is your sex drive?”, “How easily can you reach an orgasm?” and “Are your orgasms satisfying?” Items are rated on a scale from 1 (extremely) to 6 (none or never). Total scores range from 5 to 30, where 19 or greater (or 4+ per subscale) suggests sexual dysfunction. Scores are tabulated and averaged, where higher scores indicate more sexual dysfunction.

### 2.4. Data Analysis

Data were collected using Qualtrics, and all analyses were conducted using SPSS (version 29). We began by computing subscale and composite scores for each aspiration (intrinsic and extrinsic) in each domain (importance, likelihood, and attainment), averaging items to yield separate intrinsic and extrinsic goal scores per domain. To examine the relative centrality of intrinsic and extrinsic goals, we applied the relative centrality scoring method [11], which adjusts for total goal striving by controlling for the overall mean across all aspirations. This method emphasizes the qualitative balance between intrinsic and extrinsic goals, rather than the total quantity of goal pursuit, which is central to Goal Contents Theory. Assumption checks included histogram inspection for normality and linearity, and multicollinearity diagnostics via variance inflation factor (VIF) and tolerance values. Descriptive statistics and Cronbach’s alphas were computed for all relevant study variables to assess reliability.

Pearson correlations were then conducted to explore bivariate relations between goal variables, symptom domains (GERD, IBS, headache, sleep disturbance, sexual dysfunction), and selected demographic factors (e.g., age, subjective income). These analyses provided an initial overview of how goal dimensions relate to symptom patterns and financial/age-related factors. We then ran a series of hierarchical multiple regressions to examine the unique contribution of intrinsic and extrinsic goals to symptom outcomes. For each symptom domain, we first entered total goal score (the average across all intrinsic and extrinsic goals) in Step 1, to account for overall goal striving. In Step 2, either the intrinsic or extrinsic goal score was entered to assess its predictive value. This approach was used for each goal domain (importance, likelihood, attainment) and for each symptom separately, following prior SDT methodology [18,22,33].

Because intrinsic and extrinsic goals are positively correlated, and both are components of total goal striving, entering them hierarchically creates shared variance. Statistically, this manifests as cooperative suppression, where two correlated predictors reveal opposing, unique effects, when shared variance is partialled out (pp. 123–130, [40]). As expected, this suppression effect clarified the distinct roles of intrinsic and extrinsic motivation. To confirm this was not due to multicollinearity, we inspected semi-partial correlations and ensured all VIF values were below 2.5.

Finally, we conducted ANOVAs and exploratory moderation analyses to examine whether demographic factors (e.g., gender, education, income) influenced goal patterns in relation to symptom outcomes. Where relevant, we tested for interaction effects between goal dimensions and demographic variables to assess potential moderators of the aspiration-symptom relationship.

## 3. Results

### 3.1. Participants

Out of 645 patients who expressed interest in the study, either through in-person discussion or visible recruitment materials, 412 (63.8%) went on to complete the survey. However, 20 surveys were excluded from analysis for being incomplete (finished one scale or less), leaving 392 participants (60.8% response rate). A minimum sample size of 220 was considered sufficient based on a 20 observations per predictor rule [41]. The data were normally distributed and very few participants (approx. 5%) had any missing data. We therefore used the data that was obtained without replacing any missing values. The mean age in the sample was 42.8 years (SD = 12.7, range 18–65) and the sample was considered demographically diverse (see Table 1). (Note: We ran the analyses for intrinsic aspirations both with and without aspirations for physical health. We report the correlation matrix and regression results for the latter in Online Supplementary Materials Tables S1 and S2. The thrust of the results is similar, though some of the smaller effects became statistically non-significant in the absence of physical health aspirations.)

### 3.2. Relations Between Global Aspirations

First, we assessed the intercorrelations among the higher-order aspiration factors. As shown in Table 2, intrinsic goal importance, likelihood, and attainment were each positively correlated. A similar pattern emerged for the extrinsic goal domains. Additionally, intrinsic and extrinsic goal importance, likelihood, and attainment were positively correlated with one another. This pattern is consistent with prior research suggesting that active goal striving, regardless of content, is often correlated across domains [11]. It reinforces the value of using relative centrality scores in our analyses to account for overall levels of goal pursuit.

### 3.3. Correlations Between Demographics, Aspirations, and Functional Symptoms

Next, we assessed Pearson correlations between continuous demographic variables, aspiration domains, and functional symptoms (see Table 2). Age was positively associated with subjective financial status, as well as intrinsic and extrinsic goal attainment, and negatively associated with intrinsic and extrinsic goal importance, headache, and sleep disturbance. Subjective income was positively correlated with both intrinsic and extrinsic goal likelihood and attainment and negatively associated with all five functional symptoms: GERD, IBS, headache, sleep disturbance, and sexual dysfunction. The three intrinsic goal dimensions—importance, likelihood, and attainment—were positively interrelated and showed moderate positive associations with extrinsic goal dimensions. Intrinsic goal importance was weakly positively correlated with headache, while intrinsic goal likelihood was negatively associated with GERD, sleep disturbance, and sexual dysfunction. Intrinsic goal attainment was negatively associated with GERD and sleep disturbance. Among extrinsic goals, goal importance was weakly positively correlated with IBS, goal likelihood was negatively associated with sexual dysfunction, and goal attainment was negatively associated with GERD, sleep disturbance, and sexual dysfunction. All functional symptoms were positively inter-related, consistent with the overlapping nature of these conditions, such as GERD and IBS, or the disruptive effects of one symptom (e.g., sleep disturbance) on another (e.g., headache).

As zero-order correlations can be obscured by shared variance between the intrinsic and extrinsic aspiration scales, we focus on the coefficients in Step 2. They represent each goal type’s unique relation with symptoms after controlling for overall striving. These preliminary findings informed the following hierarchical regression analyses, assessing how the relative importance, likelihood, and attainment of intrinsic versus extrinsic goals related to GERD, IBS, headache, sleep disturbance, and sexual dysfunction.

### 3.4. Relations Between Aspirations and Functional Somatic Symptoms

As shown in Table 3, the relative importance of intrinsic goals was associated with less GERD, while extrinsic goal importance was associated with more GERD. Relative intrinsic goal importance was also marginally related to less sleep disturbance. The perceived likelihood of attaining intrinsic goals was associated with less GERD, IBS, headache, and sleep disturbance. Conversely, the perceived likelihood of attaining extrinsic goals was associated with more GERD, IBS, sleep disturbance, and sexual dysfunction. Greater relative attainment of intrinsic goals was associated with less GERD and sleep disturbance. Meanwhile, the relative attainment of extrinsic goals was associated with increased sexual dysfunction.

Together, these findings suggest that all three aspiration domains—importance, likelihood, and attainment—carry uniquely meaningful links to physical health outcomes. The aspiration-symptom pattern was most consistent for GERD and sleep disturbance, while other conditions showed more domain-specific effects. Next, we performed group-level comparisons on mean levels of intrinsic and extrinsic aspirations and then included the interaction between aspirations and demographic variables to see how the associations vary as a function of gender, age, education, SES, and religiosity.

### 3.5. Mean Differences Between Groups on Intrinsic and Extrinsic Aspirations

There was a main effect of gender on intrinsic goal importance (F (2, 390) = 6.33, *p* = 0.002) and attainment (F (2, 390) = 3.06, *p* = 0.048), with women scoring higher than men on both dimensions (MD = 0.28, SE = 0.08, *p* < 0.001; MD = 0.18, SE = 0.09, *p* = 0.04, respectively). There was also a main effect of education on intrinsic goal importance (F (2, 391) = 3.32, *p* = 0.037), where individuals with more education reported greater importance. However, post hoc tests did not achieve statistical significance. Extrinsic goal importance (F (2, 391) = 3.23, *p* = 0.041) and attainment (F (2, 391) = 3.92, *p* = 0.021) also varied as a function of education. Those with college or university degrees placed more importance on extrinsic goals (MD = 0.85, SE = 0.34, *p* = 0.036) and showed marginally higher attainment of those goals (MD = 0.72, SE = 0.33, *p* = 0.076) than those with less than high school. Religiosity was positively associated with intrinsic goal importance (F (2, 391) = 3.21, *p* = 0.041). Individuals who rated religion as “very important” scored higher on intrinsic goal likelihood than those who rated it as “not at all important” (MD = 0.34, SE = 0.14, *p* = 0.052). See Table 4 for subgroup means across the goal orientations. There were no effects for any other variables.

### 3.6. Demographic Interactions in the Links Between Aspirations and Symptoms

We next examined interactions between demographic variables and goals in relation to symptoms using between-group ANOVAs. In line with prior studies [18], goal variables were grouped into meaningful categories using median-split (e.g., higher vs. lower), allowing us to identify patterns of symptom burden across subgroups. For gender, women who reported low likelihood of attaining intrinsic goals experienced more GERD than men who reported low intrinsic goal likelihood (F (1, 297) = 5.95, *p* = 0.015). With education, individuals with less than high school who rated intrinsic goals as low in importance (F (2, 307) = 1.39, *p* = 0.093), likelihood (F (2, 290) = 1.37, *p* = 0.084), and attainment (F (2, 289) = 1.81, *p* = 0.004) experienced marginally more GERD, compared to those with high school or more. Those with less than high school also experienced more IBS (F (2, 252) = 1.58, *p* = 0.026) and sleep disturbance (F (2, 277) = 1.82, *p* = 0.006) when the likelihood of attaining intrinsic goals was rated as low. For income, compared to the moderate ($50,000–100,000/year) and high earning group ($100,000+/year), participants earning under $49,000/year who perceived low intrinsic goal likelihood (F (2, 272) = 1.45, *p* = 0.031) and low extrinsic goal attainment (F (2, 285) = 1.47, *p* = 0.032) had more GERD, as well as marginally more sleep disturbance (F (2, 266) = 1.36, *p* = 0.071) when extrinsic goal attainments were rated as low. Compared to the lowest and highest earning groups, however, participants earning $50,000–$100,000 who perceived high likelihood of attaining extrinsic goals reported more sleep disturbance (F (2, 261) = 1.41, *p* = 0.049). Lastly, religiosity moderated several associations. Participants who rated religion as “somewhat important” and perceived low intrinsic goal likelihood (F (2, 279) = 1.49, *p* = 0.024) or high extrinsic goal likelihood (F (2, 282) = 1.40, *p* = 0.051) reported more headaches, compared to those rating religion as “not at all important” or “very important”. This same group also had more headaches (F (2, 289) = 1.53, *p* = 0.021) and marginally more GERD (F (2, 287) = 1.32, *p* = 0.090), IBS (F (2, 250) = 1.41, *p* = 0.052), and sleep disturbance (F (2, 269) = 1.33, *p* = 0.088) when extrinsic goal attainment was rated as high.

## 4. Discussion

This study extends SDT by examining whether the relative prioritization of intrinsic versus extrinsic life aspirations is associated with common functional medical symptoms. Our findings broadly support the proposition that intrinsic aspirations may be protective, while extrinsic aspirations may elevate functional symptom risk, with effects varying by goal domain (importance, likelihood, attainment) and symptom type. These patterns align with Goal Contents Theory, which posits that intrinsic goals support psychological need satisfaction and health, whereas focusing on extrinsic goals may frustrate needs and contribute to stress-related outcomes.

Consistent with suppression effect theory [40], intrinsic and extrinsic goals demonstrated opposite, unique associations primarily with symptoms of GERD and sleep disturbance, after controlling for overall levels of goal striving. These findings build on prior SDT research linking intrinsic aspirations to reduced stress, healthier behavior regulation, and improved physiological outcomes [12,16,17]. Notably, this study extends that literature by examining three distinct temporal goal domains—importance, likelihood, and attainment—whereas most prior studies have focused on only one or two [33,42,43]. We also advance the field by conducting this study in a real-world primary care population—an underrepresented setting in SDT research—which enhances generalizability beyond student or convenience samples.

The risks of extrinsically oriented striving were most pronounced for GERD and sleep disturbance. Individuals who rated extrinsic goals as more important or more likely to be attained reported greater physical symptoms. This supports the view that externally driven striving—often shaped by pressure, comparison, or contingent self-worth—may dysregulate stress-response systems or interfere with circadian and regulatory processes. Prior research has linked extrinsic aspirations to somatic symptoms like headaches, gastrointestinal and musculoskeletal pain [18,19,20], but our findings add specificity by focusing on functional syndromes highly prevalent in primary care.

Furthermore, an especially novel finding was that greater endorsement of extrinsic goals was associated with more symptoms of sexual dysfunction—an underexplored area in SDT research. Although preliminary, this may reflect how externally driven pursuits, such as striving for financial success, image, or fame, could interfere with emotional intimacy, body confidence, or self-worth, which are important for healthy sexual functioning. This interpretation aligns with research showing that autonomy-supportive romantic partnerships predict better physiological regulation and lower blood pressure under stress [44], reinforcing the broader somatic relevance of motivational quality.

### 4.1. Demographic Moderation and Sociocultural Context

Exploratory moderation analyses revealed meaningful demographic patterns in the link between aspirations and outcomes. Subjective financial status was positively associated with overall goal pursuit, yet neither it nor objective income moderated aspiration-symptom associations. This aligns with foundational Goal Contents Theory studies [18,45] suggesting that the contents of one’s goals—not the quantity of financial resources per se—better predicts well-being and health. These results imply that striving may operate similarly across economic strata, reinforcing the cross-cultural relevance of Goal Contents Theory.

However, important subgroup effects emerged. For instance, women who perceived their intrinsic goals as less likely to be attained reported more GERD, compared to men with similarly low expectations. Likewise, compared to those who completed high school or college/university, participants with lower education who viewed intrinsic goals as less important, attainable, or achieved, experienced greater symptom burden across GERD, IBS, and sleep disturbance. These findings suggest that education- and gender-based vulnerabilities may lead to somatic fallout when intrinsic goals, in particular, feel unattainable. These results echo a pattern reported by Guillen-Royo and Kasser [46] in which intrinsic aspirations actually lowered happiness for participants from a Lima slum. Arguably, the unexpected effect arose because structural and systemic constraints made those goals hard to attain, suggesting that it is not intrinsic goals themselves that become harmful, but rather the frustration of being unable to realize them, as shown in our data.

Religiosity also moderated several relationships. Participants who rated religion as “somewhat important” reported more physical symptoms—especially headaches, GERD, and IBS—when extrinsic goals were prioritized, or intrinsic goals were perceived as unlikely. One speculative interpretation is that partially internalized belief systems may create motivational conflict or tension. In SDT terms, this reflects introjected motivation: a state in which behavior is driven by guilt, obligation, or conditional self-worth rather than authentic endorsement [47]. These motivational tensions may contribute to chronic stress and, in turn, somatic symptom expression.

Taken together, these findings offer preliminary support for the relevance of Goal Contents Theory, not only in understanding psychological outcomes but also in explaining common physical symptoms. Motivational profiles—particularly the balance between intrinsic and extrinsic goals—may influence symptom vulnerability through their impact on perceived control, stress reactivity, and psychological need fulfillment [48,49]. While these associations remain correlational and exploratory, they underscore the potential utility of integrating motivational assessment and support into biopsychosocial models of care. Importantly, life goals are not merely personal preferences—they are shaped by social norms, structural realities, and broader cultural contexts that must be acknowledged in both research and clinical practice.

### 4.2. Limitations and Future Research

This study has several limitations. First, it relied on self-report data and a non-random sampling procedure, which may introduce reporting biases and limit generalizability. Although the sample was socioeconomically and demographically diverse, it was drawn from a single Canadian city and predominantly comprised Caucasian participants. Future studies should replicate these analyses across broader geographic and cultural contexts.

Second, while the aspiration scales used were previously validated in general populations, they may not fully capture the cultural nuance or clinical salience of goal pursuit in all sociodemographic groups. Further, although we explored some gender and cultural moderators, future studies should more explicitly test how goal orientations vary across gender identities, ethnic backgrounds, and sociocultural belief systems. For example, gender norms may influence which aspirations feel accessible or socially sanctioned, while structural or cultural barriers may shape perceptions of goal attainability.

Third, although our hypotheses were grounded in SDT, we did not directly assess key mediating constructs, such as psychological need satisfaction, need frustration, or perceived stress. As such, we cannot draw conclusions about the causal mechanisms linking motivational orientation to physical symptom burden. Future research should incorporate multi-method designs, such as biomarker assessment, ecological momentary sampling, behavioral tracking, or qualitative interviews, to examine pathways of influence and validate these preliminary findings.

Fourth, while we explored a range of demographic moderators, several subgroup effects were marginal and should be interpreted with caution. Nonetheless, they raise important questions about how goal orientations interact with sociocultural vulnerability factors such as gender, education, income, and religiosity. Future studies should model these interactions longitudinally and consider how structural barriers and contextual supports influence the formation, pursuit, and health consequences of intrinsic versus extrinsic goals. Additionally, some associations, such as between intrinsic goal attainment and sexual dysfunction, did not reach statistical significance despite plausible theoretical links and reasonably sized effects, which may be due to reduced response rates on that outcome variable and loss of statistical power. These results should be considered inconclusive pending replication.

Finally, this study focused on five specific functional symptom domains. Although these were selected for their prevalence and relevance in primary care, future work could extend this approach to other conditions such as fatigue, chronic pain, or multi-morbidity, to test the robustness and scope of the observed associations. For instance, previous studies have shown that preventive interventions targeting motivation and coping in healthcare professionals, such as mindfulness and emotional regulation training, can improve both psychological and physical outcomes [50]. Such work may help inform strategies for addressing functional symptoms in broader clinical populations.

## 5. Conclusions

This preliminary study contributes to a growing body of research linking motivation and health by demonstrating that the relative prioritization of life goals—particularly the distinction between intrinsic and extrinsic aspirations—may relate not only to psychological outcomes but also to the experience of common physical symptoms seen in primary care. Individuals who placed greater importance on intrinsic goals and viewed them as attainable reported fewer symptoms of GERD and sleep disturbance. Conversely, individuals who prioritized extrinsic goals—especially in terms of likelihood and attainment—reported more symptoms of GERD, sleep disturbance, and sexual dysfunction. Importantly, these associations were shaped by sociodemographic context, with differential risk patterns observed across gender, education, and religiosity.

By integrating Goal Contents Theory into a primary care setting, this study underscores the clinical relevance of personal goals in biopsychosocial care. While further research is needed to confirm these findings and clarify underlying mechanisms, this work suggests that assessing and supporting patients’ intrinsic life goals may represent a promising, underutilized lever for promoting well-being and reducing symptom burden, particularly for conditions rooted in stress and lifestyle. Attending to the motivational landscape of patients may not only enrich individualized care but also bridge a longstanding gap between psychological theory and everyday medical practice.

## Figures and Tables

**Table 1:** Sample Characteristics (N = 392).

Factor	Category	Frequency (%)
Gender	Woman	198 (50.5)
	Man	192 (49.0)
	Non-binary	2 (0.5)
Education	Lower than high school	11 (2.8)
	High school	86 (21.9)
	College/University or higher	295 (75.3)
Income	$49,000 and under	112 (28.6)
	$50,000 to $99,000	161 (41.1)
	$100,000 and over	118 (30.1)
Subjective financial status	1–5 (bottom half) 6–10 (top half)	121 (18.1) 271 (81.9)
Ethnicity	White	322 (82.1)
	Black	3 (0.8)
	Latino or Hispanic	9 (2.3)
	Asian	33 (8.4)
	Native Hawaiian/Pacific Islander	1 (0.4)
	Indigenous	3 (0.8)
	Other	22 (5.6)
Religiosity	Not at all important	233 (59.4)
	Somewhat important	106 (27.0)
	Very important	53 (13.5)

**Table 2:** Correlations Between Demographic, Aspiration, and Functional Symptom Variables.

Variables	1	2	3	4	5	6	7	8	9	10	11	12	13
1. Age	—												
2. Sub inc.	0.21 **	—											
3. Int imp	−0.16 **	0.09	—										
4. Int lik	−0.06	0.31 **	0.62 **	—									
5. Int att	0.13 **	0.36 **	0.41 **	0.76 **	—								
6. Ext imp	−0.14 **	0.08	0.28 **	0.21 **	0.12 *	—							
7. Ext lik	−0.07	0.33 **	0.36 **	0.55 **	0.46 **	0.66 **	—						
8. Ext att	0.19 **	0.44 **	0.25 **	0.48 **	0.58 **	0.45 **	0.78 **	—					
9. GERD	−0.04	−0.28 **	−0.08	−0.26 **	−0.23 **	0.04	−0.09	−0.14 **	—				
10. IBS	−0.09	−0.18 **	0.06	−0.10	−0.08	0.13 *	−0.03	−0.05	53 **	—			
11. HA	−0.26 **	−0.22 **	0.13 *	−0.06	−0.04	0.08	−0.02	−0.08	0.40 **	41 **	—		
12. SLD	−0.12 *	−0.27 **	0.03	−0.21 **	−0.19 **	0.09	−0.07	−0.12 *	0.66 **	0.64 **	0.47 **	—	
13. SXD	0.04	−0.16 **	−0.02	−0.18 **	−0.11	−0.09	−0.20 **	−0.21 **	0.15 *	0.28 **	0.24 **	0.31 **	—
Mean	42.8	6.3	5.9	5.2	4.6	3.7	3.6	3.5	1.5	2.0	47.1	26.2	3.2
Std. Dev.	12.7	1.9	0.8	0.9	0.9	1.1	1.1	1.1	0.6	0.8	9.0	6.6	1.5

Sub inc., subjective income; Int imp, intrinsic aspiration importance; Int lik, intrinsic aspiration likelihood; Int att, intrinsic aspiration attainment; Ext imp, extrinsic aspiration importance; Ext lik, extrinsic aspiration likelihood; Ext att, extrinsic aspiration attainment; GERD, gastroesophageal reflux disease; IBS, irritable bowel syndrome; HA, headache; SLD, sleep disturbance; SXD, sexual dysfunction. * *p* < 0.05. ** *p* < 0.01.

**Table 3:** Regression Results for Effects of Intrinsic and Extrinsic Aspirations on Functional Symptoms and Conditions.

Variables	GERD	IBS	HA	SLD	SXD
Importance					
Step 1					
Overall	−0.02 (−0.16 to 0.11)	0.11 * (0.00 to 0.28)	0.13 ** (0.04 to 0.32)	0.09 * (−0.02 to 0.28)	−0.08 (−0.29 to 0.05)
Step 2					
Intrinsic	−0.17 ** (−0.39 to −.02)	−0.06 (−0.28 to 0.12)	0.07 (−0.10 to 0.28)	−0.09 (−0.31 to 0.07)	0.08 (−0.09 to 0.25)
Extrinsic	0.16 * (0.02 to 0.30)	0.13 (−0.06 to 0.28)	−0.09 (−0.24 to 0.08)	0.05 (−0.12 to 0.20)	−0.01 (−0.23 to 0.21)
Likelihood					
Step 1					
Overall	−0.19 *** (−0.34 to −0.11)	−0.08 (−0.21 to 0.03)	−0.04 (−0.16 to 0.07)	−0.16 *** (−0.31 to −0.06)	−0.21 *** (−0.34 to −0.10)
Step 2					
Intrinsic	−0.39 *** (−0.62 to −0.20)	−0.22 * (−0.48 to −0.03)	0.14 (−0.36 to 00.08)	−0.32 *** (−0.56 to −0.12)	−0.01 (−0.24 to 0.22)
Extrinsic	0.25 *** (0.06 to 0.41)	0.14 (−0.07 to 0.32)	0.04 (−0.14 to 0.22)	0.22 ** (0.02 to 0.38)	−0.07 (−0.29 to 0.15)
Attainment					
Step 1					
Overall	−0.21 *** (−0.36 to −0.13)	−0.07 (−0.20 to 0.04)	−0.06 (−0.19 to 0.05)	−0.17 *** (−0.33 to −0.08)	−0.15 *** (−0.27 to −0.04)
Step 2					
Intrinsic	−0.28 ** (−0.54 to −0.04)	−0.14 (−0.44 to 0.14)	0.08 (−0.19 to 0.37)	−0.23 ** (−0.54 to −0.01)	−0.18 (−0.10 to 0.47)
Extrinsic	0.08 (−0.09 to 0.24)	0.02 (−0.17 to 0.20)	−0.10 (−0.26 to 0.07)	0.05 (−0.12 to 0.21)	0.26 *** (0.06 to 0.46)

Aspiration scores for importance, likelihood, and attainment are entered hierarchically, yielding standardized regression coefficients; numbers in brackets represent 95% confidence intervals; GERD, gastroesophageal reflux disease; IBS, irritable bowel syndrome; HA, headache, SLD, sleep disturbance; SXD, sexual dysfunction. * *p* < 0.10, ** *p* < 0.05, *** *p* < 0.01.

**Table 4:** Means and Standard Deviations of Goal Orientations Across Gender, Education, Income, Ethnicity, and Religiosity.

Demographic Variable	Group	Intrinsic Importance M (SD)	Intrinsic Likelihood M (SD)	Intrinsic Attainment M (SD)	Extrinsic Importance M (SD)	Extrinsic Likelihood M (SD)	Extrinsic Attainment M (SD)
Gender	Men	5.82 (0.81)	5.17 (0.95)	4.55 (0.92)	3.63 (1.07)	3.62 (1.02)	3.44 (1.06)
	Women	6.10 (0.78)	5.35 (0.95)	4.73 (0.84)	3.76 (1.17)	3.65 (1.10)	3.47 (1.11)
Education	<High school	5.58 (0.86)	5.05 (0.79)	4.56 (0.92)	2.90 (1.19)	3.31 (0.76)	2.81 (1.00)
	High school	5.82 (0.86)	5.12 (0.96)	4.52 (0.90)	3.64 (1.20)	3.58 (1.15)	3.28 (1.07)
	College/University	6.02 (0.78)	5.31 (0.95)	4.69 (0.88)	3.75 (1.08)	3.67 (1.04)	3.53 (1.07)
Income	<$49,000/year	5.97 (0.84)	5.02 (1.04)	4.37 (0.92)	3.52 (1.29)	3.37 (1.13)	3.04 (1.16)
	$50,000–100,000/year	5.91 (0.87)	5.28 (0.99)	4.69 (0.93)	3.68 (1.13)	3.54 (1.01)	3.38 (0.99)
	>$100,000/year	5.48 (0.74)	5.48 (0.74)	4.85 (0.72)	3.70 (1.12)	4.02 (0.96)	3.94 (0.91)
Ethnicity	Asian	6.13 (0.90)	5.22 (1.16)	4.54 (1.19)	4.19 (1.03)	3.78 (0.81)	3.61 (1.01)
	Black	6.04 (0.26)	4.96 (0.38)	4.50 (0.25)	3.27 (1.08)	2.89 (0.85)	2.56 (1.08)
	White	5.92 (0.80)	5.24 (0.94)	4.64 (0.87)	3.64 (1.13)	3.62 (1.07)	3.44 (1.07)
	Hispanic	6.68 (0.23)	5.67 (0.44)	4.58 (0.51)	4.09 (1.15)	4.07 (1.52)	3.76 (1.41)
	Indigenous	6.38 (0.57)	5.54 (1.28)	4.89 (1.39)	3.62 (1.41)	3.66 (1.33)	2.78 (1.46)
	Other	6.06 (0.87)	5.49 (1.06)	4.86 (0.74)	3.60 (0.98)	3.66 (1.06)	3.53 (1.21)
Religiosity	Not at all important	5.88 (0.84)	5.19 (0.94)	4.60 (0.91)	3.64 (1.11)	3.54 (1.09)	3.39 (1.10)
	Somewhat important	6.07 (0.68)	5.30 (0.92)	4.70 (0.80)	3.85 (1.08)	3.83 (1.01)	3.61 (1.02)
	Very important	6.13 (0.87)	5.52 (1.01)	4.75 (0.91)	3.66 (1.25)	3.68 (1.01)	3.46 (1.07)

M, mean; SD, standard deviation. Scores range from 1 to 7. Higher values reflect stronger goal endorsement.

## Data Availability

The data associated with this manuscript can be made available upon reasonable request.

## Supplementary Materials

The following supporting information can be downloaded at: https://www.mdpi.com/article/10.3390/ijerph22101582/s1, Table S1. Correlations Matrix with Physical Health Aspirations Removed from the Intrinsic Aspirations Variable; Table S2. Regression Results with Physical Health Aspirations Removed from the Intrinsic Aspirations Variable.

## Abbreviations

The following abbreviations are used in this manuscript:

GERD	Gastroesophageal reflux disease
IBS	Irritable bowel syndrome
SDT	Self-determination theory
SES	Socioeconomic status
