# Supplementary material for: Linking Life Aspirations to Functional Medical Conditions: A Goal Contents Theory Perspective
Source: Int J Environ Res Public Health. 2025 Oct 17;22(10):1582. doi: 10.3390/ijerph22101582 (PMC12562518; doi:10.3390/ijerph22101582)
Supplement: Supplementary file 1 [file ijerph-22-01582-s001.zip › ijerph-3825735-supplementary.pdf]

This document contains Online Supplementary Materials for the below manuscript:

Linking Life Aspirations to Functional Medical Conditions: A Goal Contents Theory Perspective

Table S1. ***Correlations Matrix with Physical Health Aspirations Removed from the Intrinsic Aspirations Variable***

Variables	1	2	3	4	5	6	7	8	9	10	11	12	13
1. Age	—												
2. Sub inc	.21**	—											
3. Int imp	-.20**	.05	—										
4. Int lik	-.06	.32**	.66**	—									
5. Int att	.17**	.37**	.46**	.79**	—								
6. Ext imp	-.14**	.08	.26**	.21**	.11*	—							
7. Ext lik	-.07	.33**	.34**	.53**	.42**	.66**	—						
8. Ext att	.19**	.44**	.22**	.48**	.58**	.45**	.78**	—					
9. GERD	-.04	-.28**	-.06	-.19**	-.16**	.04	-.09	-.14**	—				
10. IBS	-.09	-.18**	.11*	-.05	-.02	.13*	-.03	-.05	53**	—			
11. HA	-.26**	-.22**	.16**	-.03	-.02	.08	-.02	-.08	.40**	41**	—		
12. SLD	-.12*	-.27**	.07	-.17**	-.12*	.09	-.07	-.12*	.66**	.64**	.47**	—	
13. SXD	.04	-.16**	.03	-.14*	-.05	-.09	-.20**	-.21**	.15*	.28**	.24**	.31**	—
Mean	42.8	6.3	5.9	5.2	4.6	3.7	3.6	3.5	1.5	2.0	47.1	26.2	3.2
Std. Dev.	12.7	1.9	.8	.9	.9	1.1	1.1	1.1	.6	.8	9.0	6.6	1.6

Note. Sub Inc, subjective income; Int Imp, intrinsic aspiration importance; Int Lik, intrinsic aspiration likelihood; Int Att, intrinsic aspiration attainment; Ext Imp, extrinsic aspiration importance; Ext Lik, extrinsic aspiration likelihood; Ext Att, extrinsic aspiration attainment; GERD, gastroesophageal reflux disease; IBS, irritable bowel syndrome; HA, headache; SLD, sleep disturbance; SXD, sexual dysfunction. * $p < .05$. ** $p < .01$.

Table S2. ***Regression Results with Physical Health Aspirations Removed from the Intrinsic Aspirations Variable***

Variables	GERD	IBS	HA	SLD	SXD
Importance					
Step 1					
Overall	-.01 (-.11 to .10)	.14*** (.03 to .24)	.15*** (.05 to .25)	.11* (.01 to .22)	-.03 (-.15 to .09)
Step 2					
Intrinsic	-.11 (-.26 to .04)	.01 (-.15 to .17)	.12 (-.03 to .27)	-.02 (-.18 to .13)	-.30 (-.91 to .35)
Extrinsic	.16* (.02 to .30)	.13 (-.06 to .28)	-.09 (-.24 to .08)	.05 (-.12 to .20)	.16 (-.46 to .85)
Likelihood					
Step 1					
Overall	-.18*** (-.28 to -.07)	-.04 (-.15 to .06)	-.04 (-14 to .07)	-.14*** (-.25 to -.04)	-.20*** (-.33 to -.09)
Step 2					
Intrinsic	-.18* (-.38 to -.03)	-.03 (-.25 to .19)	-.01 (-.22 to .19)	-.19* (-.39 to -.02)	-.50 (-1.13 to .68)
Extrinsic	.24** (.04 to .44)	.04 (-.18 to .27)	.03 (-.17 to .24)	.22** (.02 to .44)	-.08 (-.32 to .14)
Attainment					
Step 1					
Overall	-.17*** (-.27 to -.07)	-.04 (-.16 to .07)	-.06 (-.17 to .05)	-.14** (-.24 to -.03)	-.16** (-.28 to -.04)
Step 2					
Intrinsic	-.04 (-.25 to .17)	.07 (-.16 to .30)	.15 (-.07 to .36)	-.01 (-.23 to .20)	-.36*** (-.61 to -.12)
Extrinsic	.04 (-.19 to .27)	-.09 (-.34 to .15)	-.16 (-.39 to .07)	.01 (-.22 to .25)	.37*** (.12 to .65)

Aspiration scores for importance, likelihood and attainment are entered hierarchically, yielding standardized regression coefficients; numbers in brackets represent 95% confidence intervals; GERD, gastroesophageal reflux disease; IBS, irritable bowel syndrome; HA, headache, SLD, sleep disturbance; SXD, sexual dysfunction.

* $p < .10$. ** $p < .05$. *** $p < .01$.
